# Cell Type-Specific Modulation of Respiratory Chain Supercomplex Organization

**DOI:** 10.3390/ijms17060926

**Published:** 2016-06-21

**Authors:** Dayan Sun, Bin Li, Ruyi Qiu, Hezhi Fang, Jianxin Lyu

**Affiliations:** Key Laboratory of Laboratory Medicine, Ministry of Education, Zhejiang Provincial Key Laboratory of Medical Genetics, College of Laboratory Medicine and Life Sciences, Wenzhou Medical University, Wenzhou 325035, China; dayansun0128@163.com (D.S.); zblibin123@163.com (B.L.); 15167797018@163.com (R.Q.)

**Keywords:** mitochondrial supercomplex, nuclear genetic background, respiratory chain, mitochondrial dysfunction, Leigh’s disease

## Abstract

Respiratory chain complexes are organized into large supercomplexes among which supercomplex In + IIIn + IVn is the only one that can directly transfer electrons from NADH to oxygen. Recently, it was reported that the formation of supercomplex In + IIIn + IVn in mice largely depends on their genetic background. However, in this study, we showed that the composition of supercomplex In + IIIn + IVn is well conserved in various mouse and human cell lines. Strikingly, we found that a minimal supercomplex In + IIIn, termed “lowest supercomplex” (LSC) in this study because of its migration at the lowest position close to complex V dimers in blue native polyacrylamide gel electrophoresis, was associated with complex IV to form a supercomplex In + IIIn + IVn in some, but not all of the human and mouse cells. In addition, we observed that the 3697G>A mutation in mitochondrial-encoded *NADH dehydrogenase 1* (*ND1*) in one patient with Leigh’s disease specifically affected the assembly of supercomplex In + IIIn + IVn containing LSC, leading to decreased cellular respiration and ATP generation. In conclusion, we showed the existence of LSC In + IIIn + IVn and impairment of this supercomplex causes disease.

## 1. Introduction

Mitochondria are the power plants of the cell, generating Adenosine triphosphate (ATP) through the oxidative phosphorylation (OXPHOS) system. In mammals, the OXPHOS system consists of five complexes (I to V) that are embedded in the inner mitochondrial membrane and harbor two mobile electron carriers, ubiquinone and cytochrome *c* (cyt *c*). In the electron transfer chain (ETC), electrons are transferred among these complexes generating a proton gradient across the inner mitochondrial membrane, which is used by complex V (F_1_F_0_-ATP synthase) to synthesize ATP from ADP [1]. Electrons from complexes I (NADH dehydrogenase) and II (succinate dehydrogenase) are transferred to complex III (coenzyme Q-cyt *c* reductase) via ubiquinone. Finally, complex IV (cyt *c* oxidase) receives electrons from complex III via cyt *c* to reduce molecular oxygen to water.

Historically, respiratory chain complexes have been thought to randomly diffuse throughout the inner mitochondrial membrane [2]. In the “fluid state model”, electron transfer is based on random collision of the electron carriers within the respiratory complexes. However, this model challenged the old “solid state model”, in which the respiratory complexes have been suggested to be organized into bigger super molecules [3]. In 2000, Hermann Schägger and Kathy Pfeiffer proposed the concept of “supercomplexes” or “respirasomes” after they observed the existence of In + IIIn + IVn super molecules by blue native polyacrylamide gel electrophoresis (BN-PAGE) [4]. Currently, an intermediate model put forward by Acin-Perez *et al.* (2008) in which both freely moving OXPHOS complexes (fluid state model) and supercomplexes (solid state model) co-exist in the mitochondrial membrane, is generally accepted [5,6]. It has been hypothesized that the occurrence of supercomplexes stabilizes the structure of single complexes [7,8,9] and facilitates faster and more efficient electron transfer, while limiting the generation of reactive oxygen species (ROS) [10]. However, recent evidence suggests that supercomplexes are not kinetically important for substrate channeling [11]. Thus, the role of supercomplexes in respiration remains to be fully elucidated.

BN-PAGE is a powerful technique for the analysis of respiratory chain supercomplexes, that allowed the isolation and characterization of the respiratory chain complexes from yeast (*Saccharomyces cerevisiae*) and mammalian mitochondria [4,12]. Over the last years, the organization of supercomplexes in mammalian mitochondria has been extensively documented in animal models including mouse cell lines and bovine heart tissues using structural biology methods and BN-PAGE [5,13,14]. However, the association of single complexes within the supercomplexes in mammals remains controversial. Lapuente-Brun *et al.* reported that the assembly of supercomplexes differs in mouse strains with different genetic backgrounds such as C57BL/6J, BALB/c, and 129Sv [15], and that C57BL/6J mice do not have supercomplexes In + IIIn + IVn and III_2_ + IV. In 2014, this finding was challenged by Mourier *et al.* who showed that C57BL/6J, BALB/c, and CD1 mouse cells all possess well-organized supercomplex In + IIIn + IVn [16], but not III_2_ + IV.

The organization of individual respiratory chain complexes into supercomplexes has major implications for human diseases; remodeling of the supercomplexes plays a key role in cancer-related metabolic reprogramming [17] and ROS production associated with aging [6], as well as in mitochondrial dysfunction-associated heart failure [18]. However, the composition of human respiratory chain supercomplexes has not been elucidated yet. In this study, we examined the components of respiratory chain supercomplexes in humans and mice by using multiple human and mouse cell lines.

## 2. Results

### 2.1. Respiratory Chain Supercomplexes in Humans and Mice

**Figure 1:**
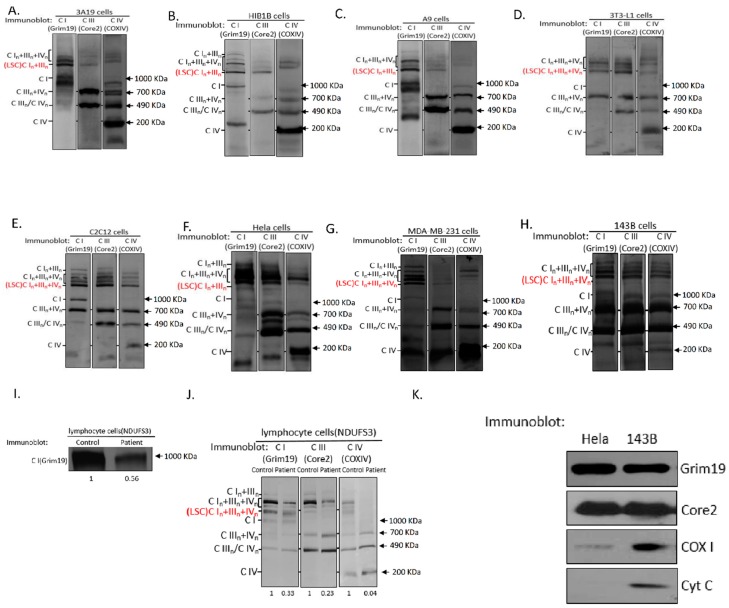
Respiratory chain supercomplexes in human and mouse cell lines. (**A**–**H**) BN-PAGE and western blot analysis of mitochondrial lysates from digitonin-solubilized 3A19 (**A**), HIB1B (**B**), A9 (**C**), 3T3-L1 (**D**), C2C12 (**E**), HeLa (**F**), MDA-MB-231 (**G**), and 143B (**H**) cells. The blots were probed with anti-Grim19, anti-Core2, and anti-COX IV; (**I**) BN-PAGE and Western blot analysis of individual OXPHOS complexes prepared with DDM. Blots for samples from control (**left**) and patient 3 (**right**) were probed with anti-Grim19; (**J**) BN-PAGE and Western blot analysis of digitonin-treated mitochondrial protein. Blots for samples from control (**left**) and patient 3 (**right**) were probed with anti-Grim19, anti-Core2, and anti-COXIV; (**K**) SDS-PAGE and Western blot/analysis of the LSC band excised from the gel after BN-PAGE. Blots were probed with anti-Grim19, anti-Core2, and anti-COX1, and anti- cyt c, respectively. In (**A**–**K**): *n* = 3. CI: complex I; CIII: complex III; CIV; complex IV.

To clarify whether the nuclear genetic background affects the formation and composition of supercomplexes, we investigated supercomplex organization in various human and mouse cell lines by standard BN-PAGE and subsequent immunoblotting. We found that cybrid 3A19 cells with the C57BL nuclear genetic background contain both IIIn + IVn and In + IIIn + IVn supercomplexes (Figure 1A). We further confirmed that C57BL/6J mice with short form of *cyt c oxidase subunit 7a polypeptide 2-like gene* (*COX7a2l*) contain both IIIn + IVn and In + IIIn + IVn supercomplexes (Figure S1A,B). Western blot analysis of mitochondrial proteins of HIB1B, A9, 3T3-L1, and C2C12 cells from Swiss Webster, C3H/An, Swiss albino, and C3H mouse strains, respectively, revealed the presence of complex IV-containing IIIn + IVn and In + IIIn + IVn supercomplexes in the different cell types (Figure 1B–E and Table S1). Similarly, complex IV-containing supercomplexes were detected in HeLa, MDA-MB-231, and 143B human cells (Figure 1F–H).

Currently, it is generally accepted that respiratory chain supercomplexes with molecular weights greater than that of complex V dimers correspond to In + IIIn and In + IIIn + IVn. Figure S1A clearly indicates that the lowest supercomplex (LSC) is composed of In + IIIn in C57BL/6J mice, while complex IV was not detected. Interestingly, we found that the 3T3-L1 and C2C12 mouse cells (Figure 1D,E) and MDA-MB-231 and 143B human cells (Figure 1G,H) did not have In + IIIn at the LSC position. Instead, In + IIIn + IVn was detected in these cell lines. To exclude potential artifacts associated with the use of detergents, we confirmed the existence of the LSC In + IIIn + IVn in C2C12 and 143B cells treated with different digitonin/protein ratios of 4, 6, and 8 g/g (Figure S1C,D). Additionally, 143B cells with different mtDNA backgrounds—B4, D4, and F2—showed the same pattern of supercomplexes, and the composition of the LSC was In + IIIn + IVn in all cell lines, indicating that the mtDNA background itself does not affect the supercomplex organization (Figure S1E).

In HeLa cells and mouse 3A19, HIB1B, and A9 cells, In + IIIn was detected at the LSC position (Figure 1A–C). Using 2D BN/SDS-PAGE, we confirmed that complex IV was associated with In + IIIn at the LSC position in protein lysates from 143B cells when compared to those from HIB1B cells (Figure S2A,B). Notably, although both the A9 and C2C12 cell lines originate from the mouse strain C3H, they exhibited a different LSC composition (Table S1). In addition, A9 and C2C12 cells displayed numerical chromosome variations; A9 was found to be aneuploid, while C2C12 was euploid [19,20]. Similarly, the nuclear genetic backgrounds of HIB1B and 3T3-L1 were different as they originated from outbred and inbred Swiss mouse strains, respectively [21,22]. To test whether culture conditions can affect the LSC organization, we cultured 143B and HeLa cells in medium containing 10 mM galactose instead of glucose. As shown in Figure S1F,G, the composition of the LSCs in both 143B and HeLa cells was not affected. Furthermore, co-migration of the LSCs from HeLa (In + IIIn) and 143B cells (In + IIIn + IVn) were detected by separating the mitochondrial complexes through BN-PAGE in the same gel. Taken together, these results suggested that the organization of supercomplex associated are cell type specific.

To investigate whether the In + IIIn + IVn supercomplex is present in different cell types, lymphoblastoid cell lines from three healthy subjects were immortalized. As shown in Figure S3, BN-PAGE showed the co-migration of complexes I, III, and IV. Strikingly, most In + IIIn + IVn was found at the LSC position in the immortalized lymphoblastoid cell lines. Therefore, we speculated that the LSC composed of In + IIIn + IVn is functionally important in mitochondria. Notably, in the lymphoblastoid cell line from patient 3, with a 595C>T mutation in the *Nicotinamide adenine dinucleotide (NADH) dehydrogenase [ubiquinone] iron-sulfur protein 3 gene* (*NDUFS3*; R199W transition at protein level), a defect of complex I impaired the assembly of complexes I, III, and IV at the LSC position as compared to those of control cells (Figure 1I,J), indicating the existence of the In + IIIn + IVn LSC. It was reported that the ratio of complex I/complex III in In + IIIn LSC was 1:2 [5]. While the molecular weights of In + IIIn LSC and In + IIIn + IVn LSC are similar, it is more likely that the ratio of complex I/complex III/complex IV in In + IIIn + IVn LSC is 1:1:1.

Cyt *c* has been demonstrated to co-migrate with supercomplex In + IIIn + IVn [5,23]. To determine whether the In + IIIn + IVn LSC contains cyt *c*, LSC proteins were excised from the BN gel and were separated with a second-dimension SDS-PAGE. As shown in Figure 1K, the LSCs of both 143B and Hela cells contained complexes I and III. Complex IV was found in 143B cells; however, it was barely detected in Hela cells (Figure 1K). In addition, cyt *c* was found only in the LSC of 143B cells.

### 2.2. Dynamics of Respiratory Chain LSC

The dynamics of respiratory chain supercomplexes, in which an 830-kDa complex I intermediate constitutes the supercomplex assembly intermediate, have been previously described [24]. After the addition of complexes III and IV, the NADH dehydrogenase module of complex I is incorporated to finish supercomplex assembly. In this model, single complex I without NADH dehydrogenase never gets fully assembled and activated when it binds to other respiratory chain complexes. These findings indicated the significance of the supercomplexes for respiratory chain function, and that altered LSC dynamics can probably affect mitochondrial function.

**Figure 2:**
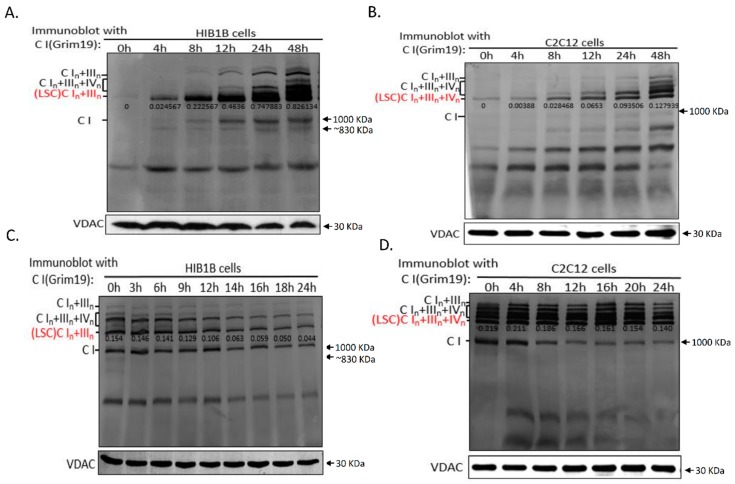
Dynamics of the respiratory chain LSC. (**A**) HIB1B and (**B**) C2C12 cells were treated with 40 µg/mL chloramphenicol (CAP) for 4–5 days; and cell pellets were collected after drug removal at 0, 4, 8, 12, 24, and 48 h. BN-PAGE and Western blot analysis of whole-cell lysates from digitonin-solubilized cells. The blots were probed with anti-Grim19. The integrated optical density (IOD) of each band was determined and is indicated; (**C**) HIB1B and (**D**) C2C12 cells were treated with CAP for 24 h. BN-PAGE and Western blot analysis of digitonin-treated whole-cell lysates. Blots were probed with anti-Grim19. Results are representative of three independent experiments.

To determine the assembly of the different complexes within the LSCs, we depleted the respiratory complexes and supercomplexes in HIB1B and C2C12 cells by treating them with chloramphenicol (CAP), which reversibly inhibits mitochondrial translation but does not affect mtDNA copy number (Figure S4). The cells were co-cultured with CAP for 4–5 days to exhaust pre-existing respiratory chain complexes, and then, the synthesis of mtDNA-encoded OXPHOS subunits and their incorporation into respiratory chain complexes were monitored after drug removal. We observed that the assembly of the LSC in both the HIB1B (In + IIIn) and C2C12 (In + IIIn + IVn) cells started as early as 4 h after drug removal (Figure 2A,B). HIB1B cells showed an approximately 32-fold increase in LSC content between 4 and 48 h after drug removal. In contrast, in C2C12 cells, a 25-fold increase in LSC content was observed during this period. The lower prevalence of LSC assembly in C2C12 cells at 48 h in C2C12 cells was confirmed by monitoring complex III-containing LSC assembly in both C2C12 and HIB1B cells (Figure S5A,B). The results corroborated that the assembly of complex IV-containing LSC was slower than that of LSC without complex IV.

Monitoring of the disassembly of the respiratory chain complexes during CAP treatment showed that disassembled LSC in HIB1B cells was 71.18% of total LSC after 24-h treatment with CAP (Figure 2C). In C2C12 cells, the disassembled portion (36.07%) was substantially lower than that in HIB1B cells (Figure 2D). We confirmed that the disassembled portion of LSC in C2C12 cells was lower than that in HIB1B cells by tracing the complex III-containing LSC (Figure S5C,D). Taken together, these observations indicated that LSCs containing IVn are more stable than those without complex IV.

### 2.3. Mitochondrial NADH Dehydrogenase Subunit 1 (MT-ND1) 3697G>A Mutation Impairs LSC Formation

To provide initial evidence that the LSC plays a role in mitochondrial biogenesis, we conducted BN-PAGE analysis of samples from patients with OXPHOS defects. The 3697G>A mutation in *MT-ND1*, which changes a highly conserved glycine to serine at the 131st residue in MT-ND1, was observed in patient 1 (Figure S6). This mutation was first identified in a patient with mitochondrial encephalopathy, lactic acidosis, and a stroke-like episode (MELAS) in 2004 [25] and was later reported in a patient with Leigh’s disease [26]; however, the pathogenic effect of the m.3697G>A mutation is not fully known. In line with a previous report [26], the mutation was found in the homoplasmic state in blood from patient 1 (total number of reads: 3322; G = 0; A = 3322), while it was not detected in blood from the patient’s mother (Figure S7). To examine the pathogenic function of the mutation, we generated cybrid cells with different m.3697G>A mutation loads. Based on the results of polymerase chain reaction-restriction fragment length polymorphism (PCR-RFLP) analysis, “L” and “H” cells with mutation loads of 0% and 96%, respectively, were selected for further analysis (Figure S8). Acquisition of private mtDNA mutations during cybrid selection in these two cell lines was excluded by whole mitochondrial genome sequencing (Table S2).

**Figure 3:**
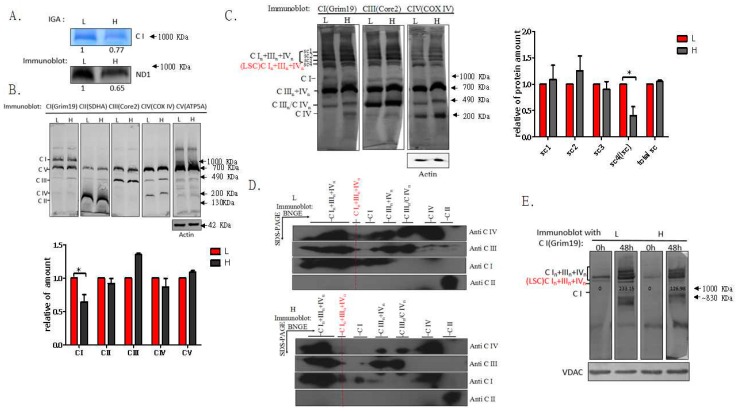
The m.3697G>A mutation impaired respiratory chain LSC. (**A**) Mitochondria from clones L and H were treated with *N*-dodecyl-β-d-maltoside (DDM) to solubilize individual OXPHOS complexes. In-gel activity assay of complex I was performed after BN-PAGE. Blots of mitochondrial protein prepared with DDM from clones L and H were probed with anti-ND1 antibody; (**B**) Total mitochondrial protein from clones L and H were solubilized with DDM (2.5 g/g protein) and subjected to BN-PAGE and Western blot analysis. The blots were probed with anti-Grim19, anti-SDHA, anti-Core2, anti-COXIV, and anti-ATP5A, respectively; (**C**) Total mitochondrial protein from clones L and H was solubilized with digitonin (6 g/g protein) and subjected to BN-PAGE and immunoblot analysis. Blots were probed with anti-Grim19, anti-Core2, and anti-COXIV, respectively; (**D**) Total mitochondrial protein from clones L and H was solubilized with digitonin and subjected to 2D BN/SDS-PAGE and Western blotting. The blots were probed with anti-Grim19, anti-SDHA, anti-Core2, and anti-COX IV, respectively. Succinate dehydrogenase complex, subunit A (SDHA) was used as a loading control; (**E**) L and H cells were treated with chloramphenicol (CAP) for three days, followed by culture without CAP for 48 h. BN-PAGE and Western blot analysis of whole-cell lysates from digitonin-treated cells. The blots were probed with anti-Grim19. In (**A**–**C**), data are presented as the mean ± SD of three replicates; in (**D**): *n* = 2; because of large SDs, results in (**E**) are representative of three independent experiments. * *p* < 0.05.

Next, we evaluated whether the m.3697G>A mutation led to impaired assembly of complex I. Total mitochondrial protein isolated from L and H cells was separated by BN-PAGE for immunoblot analysis of complex I with antibody against MT-ND1 or NADH dehydrogenase (ubiquinone) 1 alpha subcomplex 13 (NDUFA13, also termed Grim19). As shown in Figure 3A,B, H cells contained less intact complex I than L cells. However, the assembly of complexes II, III, IV, and V did not differ between L and H cells (Figure 3B). In addition, the subunit peptides of complexes I, II, III, IV, and V were not different between both cell lines (Figure S9). Since a previous study showed that most of complex I (86%) was incorporated into supercomplexes [27], we checked whether the m.3697G>A mutation impaired mitochondrial function through aberrant supercomplex formation. We determined the I-, III-, and IV-containing supercomplexes by BN-PAGE analysis of digitonin-solubilized mitochondrial samples. Interestingly, the amount of In + IIIn + IVn LSC was significantly decreased in H cells as compared to L cells, while the amount of other supercomplexes was not affected (Figure 3C). The monomeric form of complex I was hardly detected in both L and H cells (Figure 3C). By using 2D BN/SDS-PAGE, we confirmed that the abundance of In + IIIn + IVn LSCs was much higher in L than in H cells (Figure 3D). Analysis of the LSC dynamics using CAP treatment of the cells showed faster LSC assembly in L than in H cells between 0 and 48 h after CAP removal (Figure 3E). In two cybrids derived from family of patient 2 with Leigh’s disease and harboring m.14487T>C mutation loads of 11% and 96%, we did not find any alterations in the LSC from the patient (Figure S10). Taken together, our observations demonstrated that specific mutations such as m.3697G>A can affect the assembly of the LSC, which may result in impaired function of OXPHOS. However, the effect of m.3697G>A on the formation of In + IIIn + IVn LSCs is not known.

#### The *MT-ND1* 3697G>A Mutation Induces OXPHOS Defect

**Figure 4:**
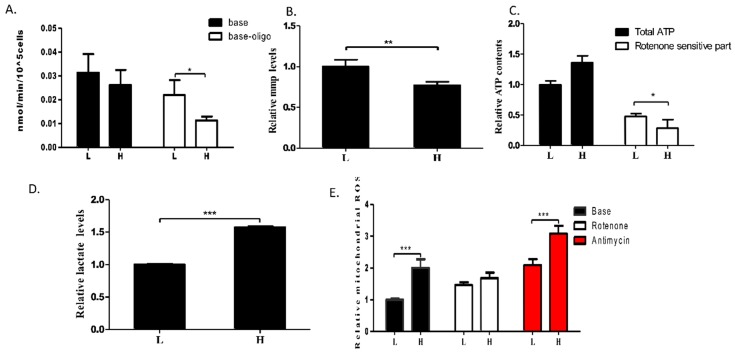
m.3697G>A mutation-induced OXPHOS defect. (**A**) Changes in mitochondrial respiratory capacity were determined in clones L and H (*n* ≥ 3). Oligomycin (2.5 µg/mL) was used to measure OXPHOS-driven mitochondrial respiration, which was calculated by subtracting the uncoupled component from basal endogenous respiration; (**B**) MMP was determined in clones L and H. MMP levels were measured in cells treated with 30 nM tetramethylrhodamine (TMRM) for 20 min (*n* ≥ 4); (**C**) Total ATP content was measured in clones L and H. Rotenone-resistant ATP content was determined in cells treated with 200 nM rotenone for 24 h. Rotenone-sensitive ATP content was calculated by subtracting the rotenone-resistant component from the total ATP content (*n* ≥ 4); (**D**) Extracellular lactate levels of clones L and H were measured in the medium after 48 h in culture (*n* ≥ 4); (**E**) Mitochondrial ROS levels were determined in clones L and H. Complex I- and complex III-related ROS generation were measured upon inhibition with 1 µM rotenone for 1 h (*n* ≥ 4) and 5 µM antimycin A for 30 min (*n* ≥ 4), respectively. The levels of MMP, ATP, lactate, and ROS were normalized against total protein concentration. For (**A**), 3–4 technical replicates were included; Error bars, ± SD; * *p* < 0.05; ** *p* < 0.01; *** *p* < 0.001.

m.3697G>A has been linked to Leigh syndrome in a few studies [26,28]. However, how m.3697G>A impairs OXPHOS function is unknown. To evaluate the mitochondrial function in cells with impaired LSC, endogenous mitochondrial respiration was measured in L and H cells. As shown in Figure 4A, although no significant decrease in base respiration was detected in intact cells, H cells showed a trend towards decreased respiration function. Further, OXPHOS-driven mitochondrial respiration was determined in L and H cells in the presence of oligomycin, which completely blocks the proton channel of ATPase. L cells exhibited higher coupled OXPHOS respiration, and consequently, higher mitochondrial membrane potential (MMP) than H cells (Figure 4A,B). These observations suggested that the m.3697G>A mutation in ND1 impairs mitochondrial function by decreasing the respiration ability of the electron transport chain.

To examine the effect of the m.3697G>A mutation on mitochondrial function further, ATP synthesis in the cells was determined. As shown in Figure 4C, although total ATP production in H cells was higher than that in L cells, ATP generation through respiratory complex I was lower in H than in L cells. Consequently, lactic acid generation was increased in the H cells due the shift of ATP generation from OXPHOS to glycolysis (Figure 4C,D). Furthermore, mitochondrial ROS production was significantly higher in the H than in the L cells (Figure 4E). After 1-h treatment with the complex I-related ROS-inducer rotenone, ROS were induced to comparable levels in both cell lines (Figure 4E). However, in L and H cells treated with the complex III-related ROS inducer Antimycin A, the total ROS level increased in both L and H cells, remaining higher in the H cells than in the L cells. This indicated that the high ROS level in the H cells was caused by impairment of complex I.

## 3. Discussion

In this study, we demonstrated that different cell types from human subjects and various mouse strains possess the respiratory chain supercomplexes IIIn + IVn and In + IIIn + IVn. However, the composition of the LSC was variable in different cell types. We found that the In + IIIn + IVn LSC is functionally important in various human and mouse cells, which is in contradiction with previous reports that In + IIIn is the only possible conformation of the LSC. The biological importance of the In + IIIn + IVn LSC was confirmed in different human mitochondrial disease models.

By BN-PAGE, some of the complexes in the OXPHOS system have been found to be organized into super molecular structures [4,12]. The existence of two major supercomplexes, In + IIIn and In + IIIn + IVn, has been widely described [5,6,15,16,29]. Further, In + IIIn + IVn is the only supercomplex with the ability to respire [5]. Although In + IIIn cannot respire, it was proposed to serve as an essential assembly intermediate of supercomplex In + IIIn + IVn [6]. Our observation that LSC assembly of In + IIIn in HIB1B cells was faster than that of In + IIIn + IVn in C2C12 cells supports the idea that In + IIIn + IVn is assembled by the addition of complex IV onto In + IIIn.

A few supercomplex assembly factors (SCAFs) for In + IIIn + IVn have been identified [15,29,30,31]. Among these, respiratory supercomplex factors 1 and 2 (Rcf1/2) are not considered real SCAFs because depletion of IIIn+IVn in Rcf1/2-null yeast mutants was caused by impaired complex IV assembly [6]. Therefore, the human cyt *c* oxidase subunit 7A-related protein (COX7RP (COX7a2l in mice]) may be the only true SCAF identified until date [15,29]. After the first description of COX7RP by Ikeda *et al.*, Lapuente-Brun *et al.* found that various mouse strains including C57BL/6J, BALB/c, and 129sv had different isoforms of COX7a2l [15,29]. Consistent with the findings of Ikeda *et al.*, Lapuente-Brun *et al.* showed that C57BL/6J and BALB/c mice with a short form of COX7a21 are not able to generate In + IIIn + IVn and IIIn + IVn supercomplexes, suggesting variable supercomplex organization in mice with different genetic backgrounds. We found that In + IIIn + IVn structure and activity were conserved in a cell line and liver tissue from C57BL/6J mice. Our results were in partial agreement with those from a recent study, in which supercomplex In + IIIn + IVn was found in C57BL/6J mouse, and its assembly seemed to be independent of COX7a2l [16]. However, our results do not exclude a role for COX7a21 in In + IIIn + IVn assembly as previously reported [15,16]. Both of these studies were focused on the long and short forms of COX7a21, but in none of them the effect of replacing one form with the other on assembly of In + IIIn + IVn was checked [15,16,29]. We hypothesize that both long and short forms of the protein may be functionally important in the assembly of the supercomplex. Future research is needed to examine the impact of COX7a21 on supercomplex assembly at the mechanistic level.

Based on their findings, Lapuente-Brun *et al.* warned for potential serious implications for studies of metabolic processes due to defects in respiratory chain activity in some laboratory mouse strains. Therefore, in this study, we conducted BN-PAGE and Western blot analysis using cell lines from multiple commonly used mouse strains. Together with previous studies [15,16], we suggested that all common mouse strains including BALB/c, CBA, CD1, 129, C3H, C57BL/6J, and inbred and outbred Swiss mice contain supercomplex In + IIIn + IVn.

Supercomplex In + IIIn is another major supercomplex in mammalian cells. Currently, the structure of In + IIIn is well characterized; however, little is known about its biological function [11,32]. Although the structure and occurrence of this supercomplex have been confirmed in multiple species such as *Arabidopsis thaliana*, *Zea mays*, and *Bos taurus* [14,33,34], we found that In + IIIn LSC occurs in some, but not all cell lines derived from various mouse strains and human subjects. Cells without In + IIIn were found to contain In + IIIn + IVn as the LSC. The occurrence of supercomplex In + IIIn + IVn as the LSC in 143B cells was first reported by Moreno-Lastres *et al.* [24]. In their work, complex IV-containing supercomplexes in wild-type 143B cells co-localized with complex I and complex III at the LSC position, while mutant 143B cells lacking complex IV did not contain the fully assembled LSC but accumulated supercomplex In + IIIn. In line with these findings, this study showed an interaction between complex IV and In + IIIn at the LSC position in 143B cells. Furthermore, the number of complex III structures in LSC with complex IV is probably lower than that in LSC without complex IV, while disruption of complex IV-containing LSC generates a supercomplex In + IIIn with a molecular mass lower than of the LSC. In addition, complexome profiling has shown co-migration of complex I, III, and IV subunits at the 1456-kDa position in human embryonic kidney (HEK293) cells, indicating the occurrence of In + IIIn + IVn rather than In + IIIn at the LSC position (~1500 kDa) [34,35].

Recently, Lapuente-Brun *et al.* proposed a model in which complex In + IIIn can either transfer electrons from NADH to freely embedded complex IV or act as an intermediate for further assembly into the In + IIIn + IVn supercomplex [5,15]. However, Moreno-Lastres *et al.* suggested that supercomplex assembly is completed only after interaction between partially assembled complex I and multiple subunits from complexes I, III, and IV [24]. In the study of Lapuente-Brun *et al.*, an A9 fibroblast cell line from an aneuploid C3H mouse strain was used, while Moreno-Lastres *et al.* used (Caucasian) human 143B cells. Interestingly, A9 and 143B cells were found to have In + IIIn and In + IIIn + IVn supercomplexes at the position of the LSC, respectively, suggesting that 143B cells may not have intermediate complex In + IIIn for further assembly of In + IIIn + IVn like A9 cells do. The use of different cells may partially explain the discrepancy between both models [5,24].

In conclusion, we investigated the possible contribution of the LSC In + IIIn + IVn to mitochondrial function. We identified a homoplasmic m.3697G>A mutation in the *MT-ND1* gene in a patient with Leigh’s disease, which coincided with decreased LSC In + IIIn + IVn steady-state levels. Without affecting the formation of other supercomplexes, the m.3697G>A mutation, which is known to be associated with Leigh’s disease, induced decreased mitochondrial respiration and ATP production, as well as increased ROS and lactate levels. This is, to our knowledge, the first report describing the mechanism of m.3697G>A in mitochondrial disease, although the mutation in mitochondrial disease has been reported in several studies [26,28,36]. Given the functional importance of the LSC In + IIIn + IVn, in addition to our findings that the composition of the LSC is variable in different cell types, we propose that mitochondria lacking In + IIIn + IVn LSC contribute to the metabolic shift in different cell models. Although for reasons other than those put forward by Lapuente-Brun *et al.* [15], we suggest that caution should indeed be applied when using different mouse strains for metabolic studies, as differences in the composition of the LSC may have an impact on the results.

## 4. Materials and Methods

### 4.1. Characterization of Patients

Patient 1 (female, aged 9 years at the time of this report) was born into a non-consanguineous Chinese family and presented with clinical manifestation of Leigh’s disease. mtDNA analysis using next generation sequencing identified a homoplasmic m.3697G>A (p.G131S) mutation in mitochondrial *ND1 gene*.

Patient 2 (male, aged 4 years at the time of this report) was born into a non-consanguineous Chinese family and presented with clinical manifestation of Leigh’s disease. mtDNA analysis by Sanger sequencing identified a heteroplasmic (by 70%) m.14487T>C (p.M63V) mutation in mitochondrial *ND6 gene*.

Patient 3 (male, had died at 20 months at the time of this report) was born into a non-consanguineous Chinese family and presented with clinical manifestation of Leigh’s disease. Mutation analysis by whole exome sequencing identified a c.595C>T (p.R199W) mutation in the *NDUFS3 gene* (MIM 60384).

Informed consent was obtained from all subjects under protocols approved by the Ethical Committee of the Peking University First Hospital (May 2014).

### 4.2. Generation of Cell Lines and Culture Conditions

Transmitochondrial cybrids were obtained by fusion of mtDNA-less ρ0 human osteosarcoma 143B cells with platelets as described previously [37]. The transformant cybrid clones were cultured in high-glucose Dulbecco’s modified Eagle’s medium (DMEM) (Hyclone, Waltham, MA, USA) containing 10% cosmic calf serum (Gibco, Carlsbad, CA, USA).

### 4.3. DNA Analysis

For patients 1 and 2, genomic DNA were extracted by following a SDS lysis protocol as described previously [38]. The entire mtDNA genome was Sanger sequenced on using 24 previously reported pairs of mtDNA primers [39]. For patient 3, whole exome sequencing was performed using Illumina Hiseq 2000 (100 bp-paired end reads).

Mutation loads of m.3697G>A in transmitochondrial cybrids were assessed by analysis of PCR-RFLP. The sequences of the primers used in this study were as follows: forward, 5’-TACTTCACAAAGCGCCTTCC-3’; reverse, 5’-ATGAAGAATAGGGCGAAGGG-3’. PCR products were subjected to complete digestion with *Hha*I (Thermo Scientific, Waltham, MA, USA) at 37 °C. Quantification of the intensity of the bands was done by Gel-Pro Analyzer 4.0 (MediaCybernetics, Warrendale, PA, USA). Mutation loads for m.14487T>C were assessed using an allele specific amplification based real-time PCR method as described previously [40]. The sequences of the primers used in this study were as follows: mtDNA 14487T: forward, 5’-AGTATATCCAAAGACAACCATCAT-3’; mtDNA 14487C: forward, 5’-AGTATATCCAAAGACAACCATCAC-3’; reverse primer for mtDNA 14487T and mtDNA 14487C, 5’-TAATGGGGTTTGTGGGGTTT-3’.

### 4.4. mtDNA Copy Number *Quantification*

Genomic DNA was extracted using a SDS lysis protocol as described [38]. A ratio of mtDNA *versus* nDNA was generated to represent the relative amount of mtDNA copy number. The Real-Time PCR reactions were performed on a 7900HT Real-Time PCR system (Applied Biosystems, Waltham, MA, USA) using SYBR^®^ Green qPCR Mastermix (Takara, Dalian, China). The detailed mouse primers were listed below: mtDNA ND2: forward, 5’-CCTATCACCCTTGCCATCAT-3’; reverse, 5’-GAGGCTGTTGCTTGTGTGAC-3’. 18s nDNA: forward, 5’-TAGAGGGACAAGTGGCGTTC-3’; reverse, 5’-CGCTGAGCCAGTCAGTGT-3’. PCR efficiencies of these primers were tested between 90% and 110%.

### 4.5. Mitochondria Isolation from Mouse Liver and Cell Lines, Protein Preparation, Blue Native PAGE, and in-Gel Activity Assay

Mitochondria from cultured cell lines were isolated as previously described [41]. Mitochondrial membrane proteins were isolated from mitochondria with n-dodecyl-β-d-maltoside (DDM, Sigma, St. Louis, MO, USA) or digitonin (Sigma). Detergent/protein ratios were 2.5 g/g and 6 g/g for DDM and digitonin, respectively [42]. Protein (60 ug) were separated by BN-PAGE (3%–11% gel) as previously described [42]. For complex I in-gel activity assays, the gels were soaked in assay buffer (25 mg nitrotetrazolium blue (NTB; Sigma) and 10 µL of 1 mg/mL reduced nicotinamide adenine dinucleotide (NADH; Sigma) in 10 mL of 5 mM Tris/HCl (pH 7.4)) for approximately 1 h. For complex IV assays, the gels were soaked in assay buffer (50 mM NaH_2_PO_4_ (pH 7.4), 5 mg 3,3’-diamiobenzidine tetra hydrochloride hydrate (DAB), (Sigma) and 50 µM cytochrome c (Sigma)).

BN-PAGE with second dimension of Tricine-SDS-PAGE was performed as described elsewhere [42].

### 4.6. Antibodies and Immunoblotting

Immunoblotting of OXPHOS complexes was conducted as described in [40], and blotting was done with anti-ND1 (1:1000; Abcam, Cambridge, MA, USA), anti-NDUFA13 (Grim19) (1:1000; MitoSciences, Eugene, OR, USA), anti-SDHA (1:1000; MitoSciences), anti-Core2 (1:1000; MitoSciences), anti-COX IV (1:11000; MitoSciences), anti-COXI (1:11,000; MitoSciences), anti-ATP synthase subunit alpha (1:1,1000; MitoSciences), anti-β-actin (1:5000; Santa Cruz, Dallas, TX, USA) or anti-VDAC (1:1000; Cell Signaling, Danvers, MA, USA) antibodies. Integrated optical density (IOD) quantification was performed using a Gel-Pro Analyzer 4.0 (MediaCybernetics).

### 4.7. Lactate Measurement

Extracellular lactate level was measured using a fluorimetric based lactate assay kit (Amplite, Foster City, CA, USA) according to the manufacturer’s instructions. Briefly, culture medium was filtered through a 10 kDa molecular weight (*M*_W_) spin filter (Millipore, Darmstadt, Germany) and fluorescence was record using a Varioskan™ Flash Multimode Reader (Thermo Scientific, Waltham, MA, USA).

### 4.8. ROS Measurement

Mitochondrial ROS were measured according to published protocols [43]. Briefly, cells were washed in Hank’s buffered salt solution (HBSS), resuspended in HBSS containing 5 μM Mito SOX (Molecular Probes), and incubated at 37 °C for 15 min. Cells were then washed (twice) with HBSS, and fluorescence was recorded using a Varioskan Flash Multimode Reader (Thermo Scientific).

### 4.9. MMP and ATP Measurements

ATP was measured using an ATP measurement kit (Molecular Probes, Carlsbad, CA, USA) according to the manufacturer’s instructions. Complex I related ATP generatioin was measured by treating the cells with 200 nM rotenone for 24 h (Sigma). MMP was determined using the cationic fluorescent redistribution dye TMRM (Thermo Scientific) as described previously [44].

### 4.10. Oxygen Consumption

Endogenous oxygen consumption by intact cells was determined using a Clark-type oxygen electrode (Hansatech, Norfolk, UK) as described previously [20]. After recording the basal respiration, oligomycin (2.5 µg/mL) (Sigma) was added to measure the un-coupling respiration of the cells.

### 4.11. Statistical Analysis

The data are presented as the mean ± SD from three independent experiments. Means were compared using independent Student’s *t-* tests using SPSS 21.0 software (IBM). *p* < 0.05 was considered significant.

## Supplementary Materials

Supplementary materials can be found at http://www.mdpi.com/1422-0067/17/6/926/s1.

## Abbreviations

BN-PAGE	blue native polyacrylamide gel electrophoresis
ATP	adenosine triphosphate
ADP	adenosine diphosphate
mtDNA	mitochondrial DNA
MMP	mitochondrial membrane potential
ROS	reactive oxygen species
OXPHOS	oxidative phosphorylation system
cyt c	cytochrome c
ETC	electron transfer chain
NDUFS3	NADH dehydrogenase (ubiquinone) iron-sulfur protein 3
CAP	chloramphenicol
MELAS	mitochondrial encephalopathy, lactic acidosis, and stroke-like episode
NDUFA13/Grim19	NADH dehydrogenase (ubiquinone) 1 α subcomplex, 13
ND1	NADH dehydrogenase subunit 1
SCAFs	supercomplex assembly factors
Rcf1/2	respiratory supercomplex factors 1 and 2
COX7a21	cyt c oxidase subunit 7A-related protein
DDM	*N*-dodecyl-β-d-maltoside
NBT	nitrotetrazolium blue chloride
DAB	3,3’-diamiobenzidine tetra hydrochloride hydrate
BCIP	5-bromo-4-chloro-3’-indolyphosphate *p*-toluidine
TMRM	Tetramethylrhodamine methyl ester
