# Supplementary material for: Cell Type-Specific Modulation of Respiratory Chain Supercomplex Organization
Source: Int J Mol Sci. 2016 Jun 21;17(6):926. doi: 10.3390/ijms17060926 (PMC4926459; doi:10.3390/ijms17060926)
Supplement: Supplementary file 1 [file ijms-17-00926-s001.pdf]

Supplementary Materials: Cell Type-Specific Modulation of Respiratory Chain Supercomplex Organization

Dayan Sun, Bin Li, Ruyi Qiu, Hezhi Fang and Jianxin Lyu

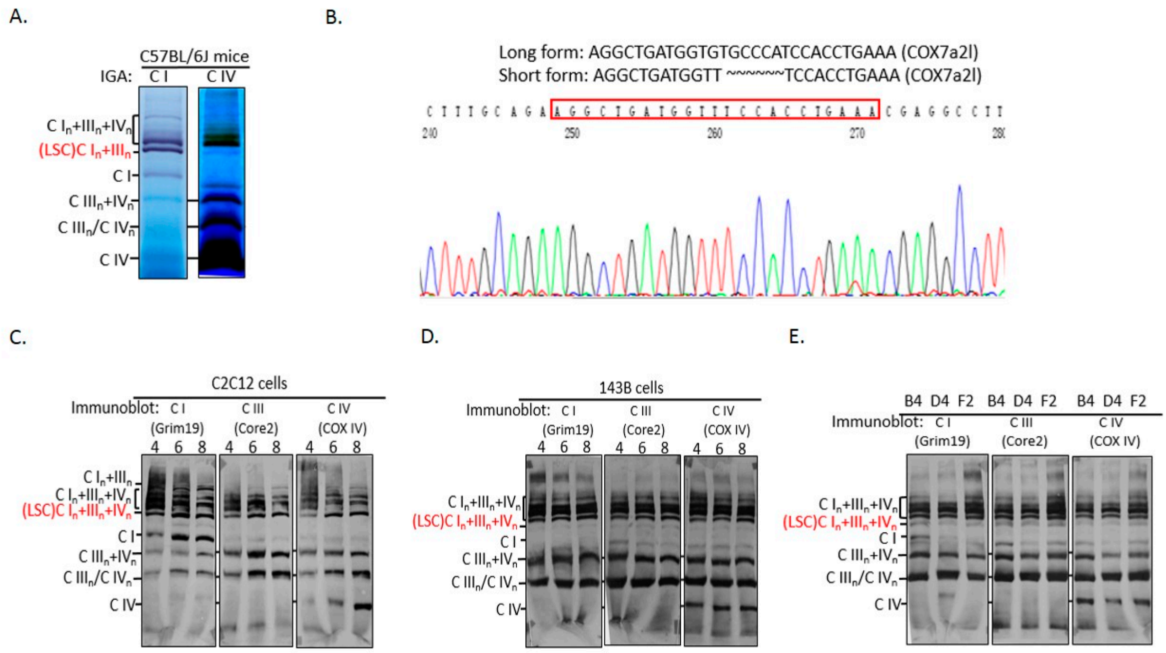

Figure S1. Organization of respiratory chain supercomplex (**A**) In-gel activity assay of complexes I and IV from the liver of C57BL/6J mice; (**B**) Sequence analysis of Cox7a2l allele in C57BL/6J mice with following primers: forward, 5′-GCTGTCTTCAGACACTCCAGAAGAGG-3′; reverse, 5′-CAAAG TGAACCAGTCCTCCACAGG-3′; (**C**,**D**) BN-PAGE/immunoblot analysis of C2C12 (**C**) and 143B cells (**D**) solubilized with digitonin at ratios of 4, 6, and 8 g/g digitonin/protein (**E**) BN-PAGE/IB analysis of mitochondrial protein extracted from 143B cybrids of three different mitochondrial DNA background (haplogroup B4, D4, and F2) with digitonin at a ratio of 6 g/g digitonin/protein. Blots were probed with anti-Grim19, anti-Core2, and anti-COX IV antibodies; Blots were probed with anti-Grim19, anti-Core2, and anti-COX IV antibodies. The LSC is indicated with a red dotted line.

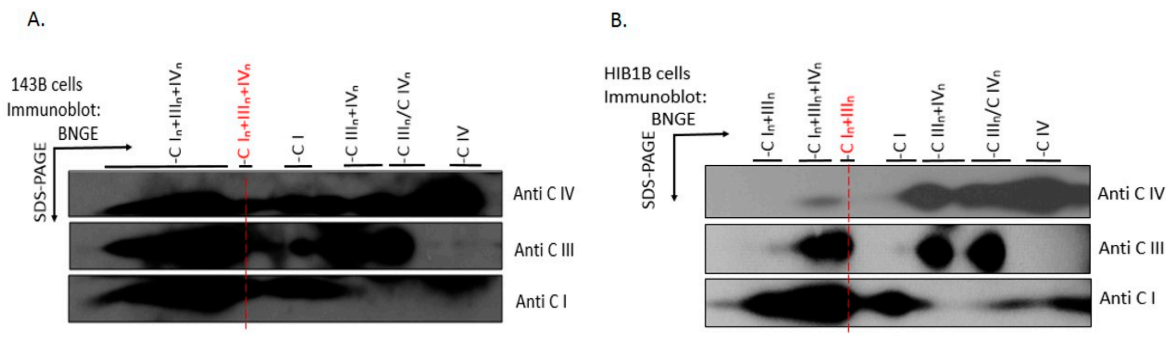

Figure S2. 2D BN/SDS-PAGE and western blotting of respiratory complexes in mitochondria prepared with digitonin from 143B cells (**A**) and HIB1B cells (**B**). The blots were probed with anti-Grim19, anti-Core2, and anti-COX IV, respectively. The LSC is indicated with a dotted line.

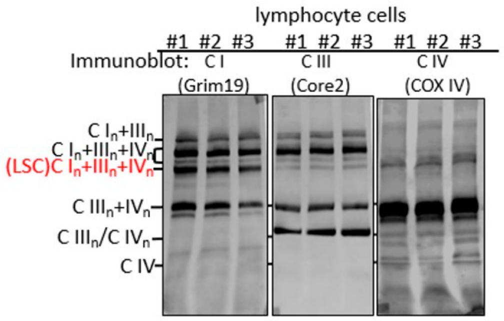

Figure S3. BN-PAGE and western blot analysis of mitochondrial protein from digitonin-permeabilized cells from immortalized lymphoblastoid cell lines derived from three healthy subjects. The blots were probed with anti-Grim19, anti-Core2, and anti-COX IV.

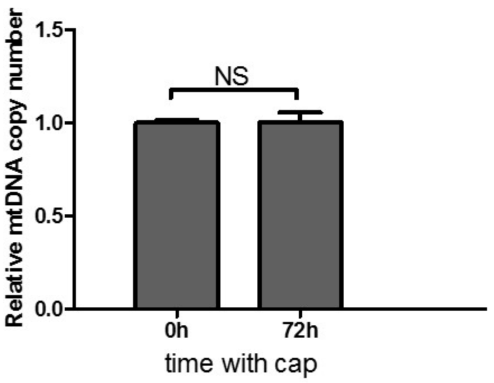

Figure S4. Relative mtDNA copy number of HIB1B cells after 0 and 72 h of treatment with chloramphenicol (CAP). NS, not significance

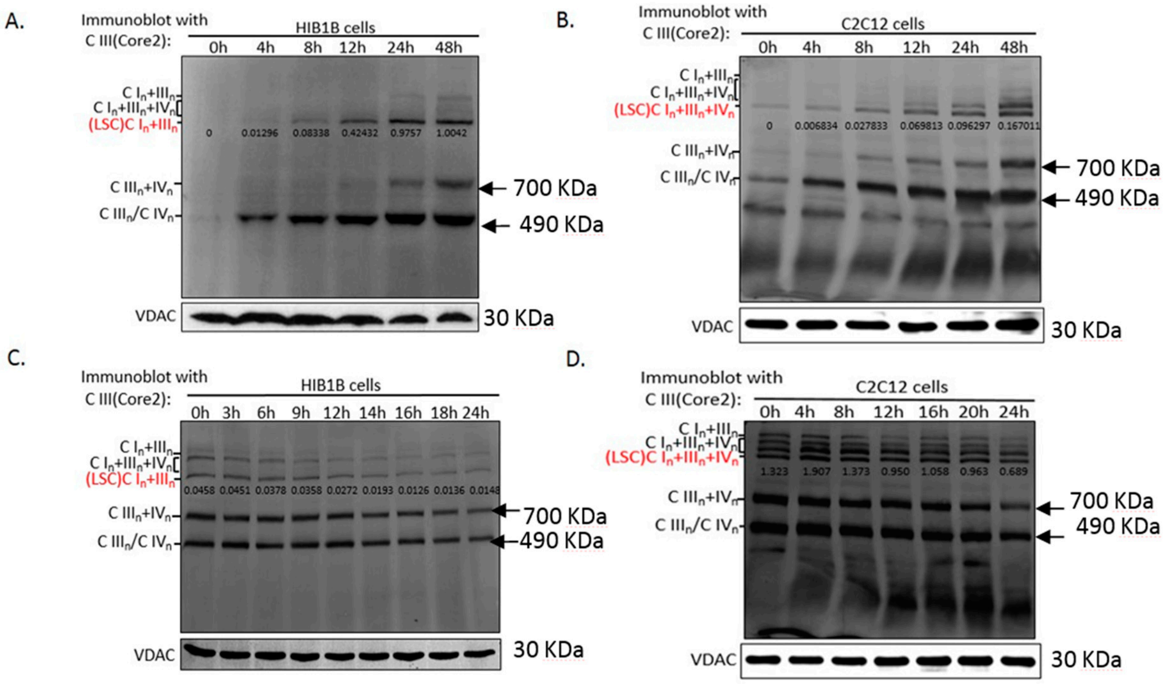

Figure S5. (**A**) HIB1B and (**B**) C2C12 cells were treated with 40 μg/mL chloramphenicol (CAP) for 4–5 days; cell pellets were collected after drug removal at 0, 4, 8, 12, 24, and 48 h. BN-PAGE and western blot analysis of whole-cell lysates from digitonin-permeabilized cells. The blots were probed with anti-Core2. The integrated optical density (IOD) of each band was determined and is indicated in the figure; (**C**) HIB1B and (**D**) C2C12 cells were treated with CAP for 24 h. BN-PAGE and western blot analysis of digitonin-treated whole-cell lysates. Blots were probed with anti-Core2. Because of large SDs, results are representative of three independent experiments.

```
                    G131S
Bos       WASNSKYALIGALRAVAQTISYEVTLAIILLSVLLMSGS
Sus       WASNSKYALIGALRAVAQTISYEVTLAIILLSVLLMNGS
Ursus     WASNSKYALIGALRAVAQTISYEVTLAIILLSVLLMNGS
Dugong    WASNSKYALIGALRAVAQTISYEVSLAIILLPTMLMNGS
Macropus  WASNSKYALIGALRAVAQTISYEVTLAIILLSIMLINGS
Tarsius   WASNSKYALIGALRAVAQTISYEVTLAIILLAILLMSGS
Gorilla   WASNSNYALIGALRAVAQTISYEVTLAIILLSTLLMNGS
Pan       WASNSNYALIGALRAVAQTISYEVTLAIILLSTLLMSGS
Cebus     WASNSNYALIGALRAVAQTISYEVTLAIILLSTLLMSGS
```

Figure S6. Conservation analysis of the m.3697G>A transition in the MT-ND1 gene (G131S substitution). G, Gly; S, Ser.

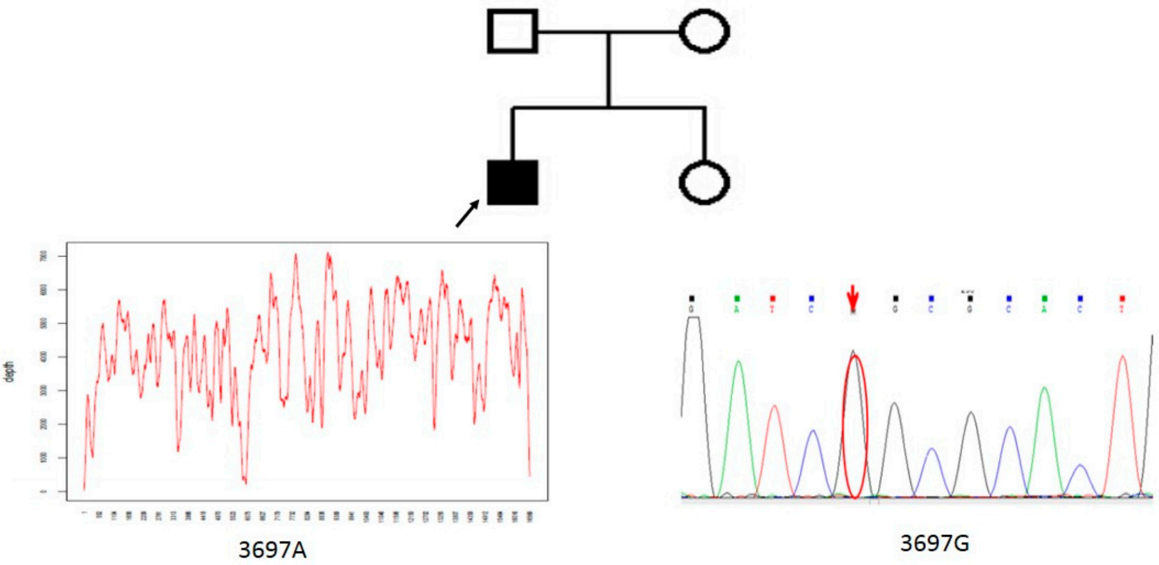

Figure S7. Next-generation sequencing of MT-ND1 gene from the blood of patient 1 (indicated by a black arrow) (total reads of 3697 in next-generation sequencing: 3322; G = 0; A = 3322). Blood from the mother of the patient were Sanger sequenced in the gene of MT-ND1 (indicated by a red arrow).

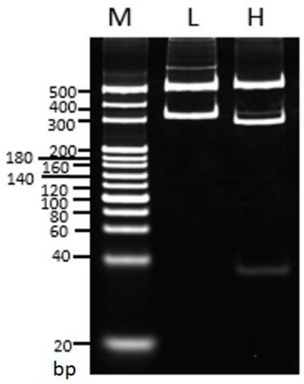

Figure S8. PCR-RFLP analysis of MT-ND1 sequences from L and H cells using *Hha*I. Fragment (832 bp) of L cells without m.3697G>A was cut into two small fragments of 528 bp and 304 bp; Fragment of H cells with a homoplasmic m.3697G>A was cut into three small fragments of 528 bp, 270 bp, and 34 bp. M: DNA marker.

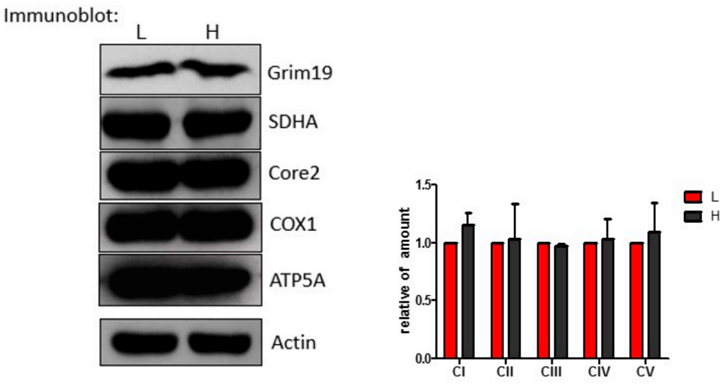

Figure S9. Whole cell of clones L and H were solubilized with RIPA buffer and subjected to SDS-PAGE and western blot analysis. The blots were probed with anti-Grim19, anti-SDHA, anti-Core2, anti-COX IV, and anti-ATP5A, respectively. Actin was used as internal control. Results were representative of three independent experiments. Error bars, ±SD.

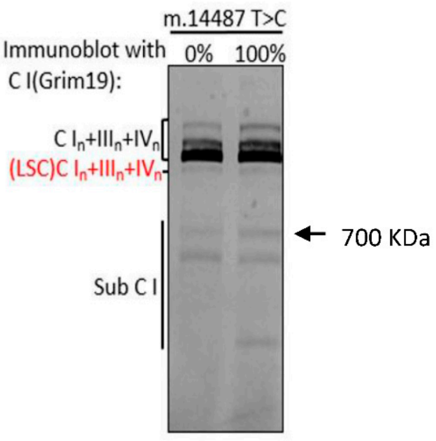

Figure S10. BN-PAGE and western blot analysis of whole-cell lysates from digitonin-treated cells. Western blots of control (**left**: 0% m.14487T>C) and patient 2 (**right**: 100% m.14487T>C) samples were probed with anti-Grim19.

Table S1. Genetic backgrounds of eight cell lines.

Cell Line	Cell Type	Strain	LSC	Ref.
A9	fibroblast (areolar and adipose)	C3H/An mouse	$I_n + III_n$	[45]
3A19	Lewis Lung	C57BL mouse	$I_n + III_n$	[20]
HIB1B	fibroblasts (brown preadipocytes)	Swiss Webster mouse	$I_n + III_n$	[22]
C2C12	myoblast (muscle)	C3H mouse	$I_n + III_n + IV_n$	[19]
3T3-L1	fibroblast (Embryo)	Swiss albino mouse	$I_n + III_n + IV_n$	[21]
Hela	Epithelial (Cervix)	Cervical cancer (African American)	$I_n + III_n$	[46]
143B	osteosarcoma cells	Osteosarcoma (Caucasian)	$I_n + III_n + IV_n$	[47]
MDA-MB-231	Epithelial (Mammary Gland)	Breast adenocarcinoma (Caucasian)	$I_n + III_n + IV_n$	[48]

LSC: lowest supercomplex; $I_n + III_n$: respiratory chain supercomplex $I_n + III_n$; $I_n + III_n + IV_n$: respiratory chain supercomplex $I_n + III_n + IV_n$.

Table S2. Analysis of whole mitochondrial genome in patient 1.

Position	Gene	rCRS Base	Mutation (L)	Mutation (H)	AA Change	mtDNA Databases *
73	D-loop	A	G	G	no	Polymorphic Sites
207	D-loop	G	A	A	no	Polymorphic Sites
263	D-loop	A	G	G	no	Polymorphic Sites
502	D-loop	G	A	A	no	Polymorphic Sites
16136	D-loop	T	C	C	no	Polymorphic Sites
16183	D-loop	A	C	C	no	Polymorphic Sites
16189	D-loop	T	C	C	no	Polymorphic Sites
16218	D-loop	T	C	C	no	Polymorphic Sites
16310	D-loop	A	G	G	no	Polymorphic Sites
16355	D-loop	C	T	T	no	Polymorphic Sites
750	12s rRNA	A	G	G	no	Polymorphic Sites
827	12s rRNA	A	G	G	no	Polymorphic Sites
1438	12s rRNA	A	G	G	no	Polymorphic Sites
1719	16s rRNA	G	A	A	no	Polymorphic Sites
2220	16s rRNA	A	G	G	no	Polymorphic Sites
2706	16s rRNA	A	G	G	no	Polymorphic Sites
2831	16s rRNA	G	A	A	no	Polymorphic Sites

Table S2. *Cont.*

Position	Gene	rCRS Base	Mutation (L)	Mutation (H)	AA Change	mtDNA Databases *
**3697**	**ND1**	**G**	**G**	**A**	**Gly>Ser**	**Pathogenic Mutation**
4769	ND2	A	G	G	no	Polymorphic Sites
4820	ND2	G	A	A	no	Polymorphic Sites
8860	ATPase6	A	G	G	Thr>Ala	Polymorphic Sites
10310	ND3	G	A	A	no	Polymorphic Sites
11719	ND4	G	A	A	no	Polymorphic Sites
13590	ND5	G	A	A	no	Polymorphic Sites
14766	Cytb	C	T	T	Ile>Thr	Polymorphic Sites
15301	Cytb	G	A	A	no	Polymorphic Sites
15326	Cytb	A	G	G	Thr>Ala	Polymorphic Sites
15535	Cytb	C	T	T	no	Polymorphic Sites
15754	Cytb	C	T	T	no	Polymorphic Sites
6023	COXI	G	A	A	no	Polymorphic Sites
6216	COXI	T	C	C	no	Polymorphic Sites
6413	COXI	T	C	C	no	Polymorphic Sites
7028	COXI	C	T	T	no	Polymorphic Sites

* databases: MITOMAP, mtDB and mtSNP; AA: amino acid.
